# What Strategies Do the Nurses Apply to Cope With Job Stress?: A Qualitative Study

**DOI:** 10.5539/gjhs.v8n6p55

**Published:** 2015-09-28

**Authors:** Rasool Eslami Akbar, Nasrin Elahi, Eesa Mohammadi, Masoud Fallahi Khoshknab

**Affiliations:** 1 Arvand International Division, Ahvaz Jundishapur University of Medical Sciences, Abadan, Iran; 2 Chronic Disease Care Research Center, Ahvaz Jundishapur University of Medical Sciences, Ahvaz, Iran; 3 Nursing Department, Medical Sciences Faculty, Tarbiat Modares University, Tehran, Iran; 4 Nursing Department, University of Social Welfare and Rehabilitation Sciences, Tehran, Iran

**Keywords:** coping, Iran, job stress, nurses, qualitative content analysis

## Abstract

**Background:**

Nursing staff encounter a lot of physical, psychological and social stressors at work. Because the adverse effects of job stress on the health of this group of staff and subsequently on the quality of care services provided by nurses; study and identify how nurses cope with the job stress is very important and can help prevent the occurrence of unfavorable outcomes.

**Objectives:**

The aim of this study was to explore the experiences of nurses to identify the strategies they used to cope with the job stress.

**Methods:**

In this qualitative study content analysis approach was used. Purposive sampling approach was applied. The sample population included 18 nurses working in three hospitals. Data collection was conducted through face to face unstructured interview and was analyzed using conventional content analysis approach.

**Findings:**

The analysis of the data emerged six main themes about the strategies used by nurses to cope with job stress, which, include: situational control of conditions, seeking help, preventive monitoring of situation, self-controlling, avoidance and escape and spiritual coping.

**Conclusions:**

Exploring experiences of nurses on how to cope with job stress emerged context-dependent and original strategies and this knowledge can pave the ground for nurses to increase self-awareness of how to cope with job stress. And could also be the basis for planning and the adoption of necessary measures by the authorities to adapt nurses with their profession better and improves their health which are essential elements to fulfill high-quality nursing care.

## 1. Introduction

Nowadays, the human resource, as the most valuable asset of the organization, is facing numerous problems. One of these problems is the job stress, which has adverse effects on the bodies and souls of persons and reduces their efficiency (Rahimi, Ahmadi, & Akhond, 2004).

According to Cooper definition, job stress is the result of interaction between individuals and the working environment and actually is every kind of physical and psychological event which could lead to physical or psychological harm and, in the long run, causes negative results in the performance of the individuals and ultimately, the organization (Akbarbegloo & Vali Zadeh, 2011). Job stress can also be described as physical and emotional consequences when there is an imbalance and disparity between job demands and the amount of control that one can impose on these demands. Thus, whenever the stress occurs, it shows that the demands have surpassed the individual resources, such as physical, emotional, economic, social or psychological (V. Lambert & C. Lambert, 2008).

Although stress is appearing in all jobs, this issue is of more concern and sensitivity in careers which deal with human health so that this is the agent responsible for 30% of diseases and absences in employees in healthcare centers that costs 300-400 million dollars annually. This figure shows just the financial side of the stress while it puts a great impact on the employees’ families and also the patients (Akberbegloo & Vali Zadeh, 2011).

Among health professionals, there are some evidences that show nursing is a stressful job (Gholamnejad & Nikpeima, 2009). In several researches which have been carried out in identifying stressors in nurses, stressors such as high pressure of work and demands in the workplace (Rahimi et al., 2004; Akbarbegloo & Vali Zadeh, 2011; Gholamnejad & Nikpeima, 2009), night shift work and exposure to threats and violence in the workplace (V. Lambert & C. Lambert, 2008), sleep and rest disturbance on holidays (Gholamnejad & Nikpeima, 2009), confronting life-threatening acute emergencies and patients with unstable condition (Rahimi et al., 2004; V. Lambert & C. Lambert, 2008), contention with the physicians and weak teamwork (Rahimi et al., 2004; Akbarbeglu & ValiZadeh, 2011), vagueness in the nursing job description (Rahimi et al., 2004; Shahsavari Isfahani, Mosallanejad, Sobhanian, Tehranineshat, & Faseleh, 2005) have been proposed.

In fact, stress is a part of modern nursing which makes chronic diseases in nurses in long term such as hypertension, asthma, etc. and affects their quality of life and increase the risk of injuries due to work. Job stress can also have a significant impact on individuality of nurses and their abilities to accomplish the tasks and in particular causes weakness in decision-making, loss of concentration, apathy, loss of motivation and anxiety. And the above mentioned factors can directly lead to absenteeism, reduced efficacy, and eventually nurses’ burnout (Moustaka, Theodoros, & Constantinidis, 2010).

Nevertheless, due to the detrimental effects of job stress on physical, psychological and social health of the nurses and subsequently on the quality and efficiency of this group in health care organizations, the study and recognition of the coping process with job stress in nurses is particularly important and this could partly help prevent adverse outcomes of the stress (Rahimi et al., 2004).

Authorities have defined coping as cognitive and behavioral attempts to control the internal and external demands in encountering the surrounding environment (V. Lambert & C. Lambert, 2008). According to Poter, coping reactions to stress include activities in the social and spiritual side of human and in fact, it is considered as calming and stabilizing agent which may help individuals in maintaining their mental peace during stressful events. Thus, all responses to an environmental event may be as important as the event itself (Ahangarzadeh Rezaei, Shams, & Saghi Zadeh, 2008), for example, people who take problem-based coping strategies to reduce stress are able to show better performance to people who adopt defensive-sensitive strategies facing with high levels of stress. Taking such approaches at daily stress, these people are able to manage occupational stress effectively (Beh & Loo, 2012).

The realities show that although several studies have been conducted with the subject of nurses’ coping with occupational stress, what is noticeable as a knowledge and research gap in this area is that these studies mainly focus on quantitative approach by using general tools of measuring coping strategies and methods, and have limited themselves only to the strategies and techniques that nurses in general select and take in public conditions to reduce their stress (e.g., Laal & Aliramaie, 2010; Veronica Makie, 2003; Chang et al., 2006; Lambert et al., 2004; Mark & Smith, 2012).

In this regard, Folkman and Moskowitz (2004) stated that qualitative narrative approaches provide an interesting alternation to inventory-based approach since many things can be achieved through questioning and subsequently the emergence of stressful events in a position including what has happened, experienced, and thought. Narrative approach helps to understand what the person has coped with, especially when the stressful event is a special event that is not mentioned by the researcher.

On the other hand Garcia (2010) in his review study, reviewed of 104 published articles carried out between 1998 and June 2009, statted about the results of their coping assessment tools and methods that the issue which has been remained as a challenge regarding coping are various tools which have been applied to assess coping methods that even in the case of construction of the standard tools to assess coping strategies for adults, adding extra open-ended questions in these tools to understand stressful factors and identify real strategies of coping are highly noticeable, since the data obtained from these questions can provide insight on how adults cope with a situation, context and particular time frame of events with the normal stressful factors, and therefore, this will make the extent clear to which the preferred strategies have been reflected in quantitative tools of assessment.

Thus, the present study was carried out with a qualitative approach and with the aim of exploring the experiences of the people in order to reveal the original and related strategies used in this field to the nurses in the case of encountering occupational stress. Thus, clarifying the present conditions of the nurses, it seems necessary to obtain strategies and make the required preparations in nurses and even the nursing students as nursing professional employees in the future to adapt more and better with the occupational stressors.

## 2. Method

### 2.1 Design and Aim

This qualitative study was conducted using the conventional content analysis approach. The aim of this study was to explore the experience of Iranian nurses regarding what strategies they use to cope with job stress.

### 2.2 Setting and Participants

In this study, 18 nurses (female=11 and male=7) who had worked in different units of 3 educational hospitals affiliated to Abadan and Jahrom Universities of Medical sciences in Iran were recruited by purposeful sampling with the maximum variation sampling to achieve variation in nurse’s gender and work experiences as well as educational levels. Inclusion criteria included the willingness to participate in this research, working as full-time nurse and having at least a bachelor’s degree or master’s degree in nursing.

### 2.3 Data Collection

Data collection was conducted through an unstructured interview. Interviews carried out based on the nurses’ experiences concerning the stress caused by work and cognitive and behavioral performance of coping with the feeling stress. In this regard, the researcher interviewed with questions such as:

What stressful situations and circumstances related to your work do you experience?

Please describe completely each work stressful experience considering the time, place, location, and factors involved in tensions.

Describe feelings and thoughts at the time of encountering stressful agent.

What were your thought or actions to reduce stress?

Also, some probing questions were asked for additional clarification of the answers given by participants. Data were collected from February 2013 to May 2015. Interviews lasted between 45 and 60 minutes and were performed in a quiet place in participants working units. After recording the interviews, they immediately were transcribed word by word and analyzed at the same time. All interviews were performed by the first author and were audio taped with the nurse’s consent. The data collection, data analysis and participant’s selection were continued until data saturation occurred and a rich description of nurse’s experiences was obtained.

### 2.4 Data Analysis

Data analysis was performed using conventional content analysis approach. Content analysis consists of advanced techniques for data processing. To analyze the data, “Framework” was used as a method of qualitative data analysis. The framework is an analytical process which involves a number of distinct, though highly interconnected stages. The five key stages in qualitative data analysis involved in ‘Framework’ are: familiarization, identifying a thematic framework, indexing, charting, mapping and interpretation (Bryman & Burgess, 2002).

During the familiarization stage, the data transcribed verbatim and each interview was read several times to gain a sense of content. In the identifying a thematic framework researchers attempt to identify the key issues, concepts and themes and sets up a thematic framework within which the material can be sifted and sorted. For each passage in indexing the researchers infer and decide on its meaning, both as it stands and in the context of the interview as a whole, and record the appropriate indexing reference. In charting headings and subheadings drawn from the thematic framework and In mapping and interpretation, researchers reviewed the charts and research notes; compared and contrasted the perceptions, accounts, or experiences and searched for patterns and connections and seek explanations for these internally within the data.

### 2.5 Ethical Considerations

To run this study and after getting the necessary permits to conduct research from Arvand International branch of Ahvaz University of Medical Sciences and obtaining references and approval from Abadan and Jahrom universities to conduct the study in affiliated hospitals from authorities, the interviewers were introduced to the participants and described the research objectives and insisted on the confidentiality of all information presented by participants and their anonymity in the entire research process. Informed consents were presented to participants and if they agreed, unstructured interviews were conducted with each of them.

### 2.6 Trustworthiness

Conformity, credibility, dependability and transferability were employed to assure various aspects of trustworthiness according to Lincoln and Guba (1985). For Conformity the researchers consider their thoughts, beliefs and previous assumptions critically regarding nurses’ coping methods with job stress and made an attempt to keep this self-critical position in the whole process of the study as far as possible. During the interview, the interviewer carried out necessary revisions applying summarizing, repeating to recognize the participants’ data. To assure credibility, the text of interviews and coding them was presented to Co-researchers. Focusing on the research objectives and trying to explore the same areas for all the participants were done during the study to assure dependability. Recruiting participants with the maximum variation of sampling and thick description of the findings helped transferability of the results.

## 3. Findings

In total, 18 nurses (male=7 and female=11) with mean 32.7 (SD ± 7) years old participated in this study.14 were married and 4 single. Considering education, 16 of whom had bachelor’s degrees in nursing and two were graduate nursing students. Other characteristics of the participants are given in Table 1.

**Table 1:** Participants’ characteristics

Variable	Number (%)
Age Distribution (Year)	
20-30	7 (38.9%)
31-40	9 (50%)
41-50	2 (11.1%)
Work Experience (Year)	
1-6	8 (44.4%)
7-12	5 (27.8%)
13-18	2 (11.1%)
19-24	1 (5.6%)
25-30	2 (11.1%)
Employment Ward	
Medical	3(16.7%)
Surgical	2(11.1%)
Emergency	3(16.7%)
Pediatrics	1(5.6%)
CCU	3(16.7%)
Post CCU	3(16.7%)
ICU	2(11.1%)
Hemodialysis	1(5.6%)
Total	18

6 major categories emerged from the data analysis about coping strategies of nurses with job stress: Situational control of conditions, seeking help, preventive monitoring of conditions, self-control, avoidance, and escape the situation, and spiritual coping.

### 3.1 Situational Controlling of Conditions

Analysis of interviews with the participating nurses showed that the nurses use varieties of ways to control job stressful situations as well as stress in which they control stressful conditions regarding the circumstances of the situation through appropriate measures within the framework of the professional duties, routine administrative procedures and individual preferences and abilities.

This interaction as a situational control strategy methods includes: immediate action to control acute condition of patients, taking immediate action to control professional errors, informing the physician and other members of the treatment team, performing physician orders, informed and transparent position, understanding and sympathizing with the patient and with the patient’s attendants, leaving work with delay to ensure and control of working conditions, time management, changing shift and taking permission to leave and take advantage of the police and the judiciary.

Participant No. 7 who worked at the coronary care unit, told the researcher about how to deal with the stress of acute condition change and prompt curative measures to control the situational conditions.

“We had a patient with bradycardia and his heart rate was 50, suddenly in reduced to 30, and then he had a cardiac arrest and we had a lot of stress. We become calm only when we take an atropine injection to the patient, because after all we took some actions”

The participant No. 9 also told about time management of works to control high-pressure conditions such as doing a lot of work and duties within the limited time shifts:

*“I try to do my work on time so that I can get them out*.

To remove the concerns and stresses related to weekly work schedule, such as the interference of work with personal and family affairs was shown up in term of situational controlling of conditions strategy as changing shift and taking a day off which reflects the experience of the participant No. 6 in this regard:

*“If I was on the evening shift and my colleague the morning shift, I change my shift with my colleague since I had to leave the hospital”* (Participant No. 6).

### 3.2 Preventive Monitoring of Situation

Exploring how nurses cope with job stress showed that coping has preventive functions in some cases. This means that nurses carry out monitoring in the form of actions such as follow-up, monitoring, checking, control, being on call, increasing the accuracy, concentration, and safety, and made attempts to prevent the situations in which tension is rising in them. Although this strategy unlike other strategies focused on the future, taking this strategy was coping with the aim of controlling current stress and seemed to include a kind of sense of control over present stressful conditions.

One of the nurses in this study expressed his experience regarding proactive monitoring of the situation through regular evaluation of patient status and ongoing actions:

*“I visit ordinary patient who did not take any hazardous medicine every two hours and the patients who took hazardous medicine every hour”* (Participant No. 4).

In some wards, such as CCU nurses conduct the kind of monitoring and control which was besides features such as predicting possible events and readiness to take the appropriate measures as soon as possible which was named on call in this study. This proactive monitoring of situations helps reduce the stress of nurses as well as increasing nurses’ readiness and dominance. Participant No. 7, who was a nurse working in the coronary care unit, told the interviewer about using this method:

*“We nurses bear always in mind that everything may happen to the patient and we should take necessary measures to reduce our stress*”.

### 3.3 Seeking Help

The findings of the study showed that, when facing work stressful situations, nurses, in some cases, use strategies in which they ask for help, take the supports of the coworkers or other staff or even the family members and try to cope with professional stressful situations. The results of this study reflect the fact that although previous strategy, i.e. situational controlling of condition and preventive monitoring of the situation, was based on self-confidence coping strategies, in seeking help strategy the nurses prefer to consciously use reliable and effective social capabilities around themselves (the staff of the institution and the family) in order to cope with job stress.

For example, seeking help from colleagues or nursing managers was manifested in stressful situations in which the nurses required informational support, or encountered challenges in their daily assigned duties and responsibilities for any given reason. The participant No. 13 expresses her experience in this regard:

*“When I have a question I do not feel ashamed and ask the supervisor or my colleagues”*.

To cope with the job stress, the nurses sometimes shared job stress problems with their families which usually give them emotional and informational supports. The participant No. 11 told the researcher:

“*When I have a job stress, I talked to my mother or my spouse”*.

### 3.4 Self-Controlling

The results of the interview with the participants showed that, to cope with job stress, the nurses used the strategies where they tried to emotionally and cognitively focus on self-control rather than stressful external conditions and increase their adaptation to the situation that disrupt the balance of these aspects. The self-control strategy were carried out by the nurses with using methods such as positive thinking, the silence, tolerance and forced acceptance, crying, self-learning, the use of recreation and sports.

Concerning positive thinking to overcome negative thoughts and feelings at the time of treatment for a patient with acute situation, participant No. 7 stated:

“*Sometime in facing acute changes in the patient, in order to calm them up, I tell me that what I did make the patient better. I tell myself do not be afraid, she gets better, wait*”.

The results also showed that nurses use recreation and sports to evacuate occupational stress and reduce the impact of work conflict and help physical and mental reconstruction. This self-control method which is based on what the nurses expressed helped them cope with the stress of the job. Participants No.14, who was a young nurse, told the researcher about his experiences on how to cope with job stress:

*“I love music, instead of thinking about the work problems at home; I try to calm myself* with *music*”.

### 3.5 Avoidance and Escape the Situations

The strategy which is done through avoidance and escape methods is appeared in the stressful situations where the nurses preferred to avoid or escape from them for reducing tensions and stress and achieving peace. Avoiding carrying out an action, declining the request of others, giving no attention to negative feelings, diverting away bad thoughts, and avoiding and escaping from stressful situations are some behaviors that nurses use in avoidance and escape coping strategy. Participants (No.10) said:

*“One thing I do when I am stressful about events that occurred in the hospital, I make me busy in home with computer or internet or do some chores to forget the problem”*.

### 3.6 Spiritual Coping

Participating nurses expressed spiritual coping as one of their strategies to cope with job stress. This approach, which was manifested as spiritual practices such as the prayer or reading the Quran, could include features such as seeking help from God and feeling support from a superior force which was taken by nurses in different work situations. And this spiritual coping, causes tension relieving in the nurses. For example, participants No.13 told the researcher In this regard:

“*Effect of spirituality is very high. Sometimes working pressure is high. It makes you tired. If there is no power of faith you feel alone also I read Quran when I leave home to go to work and it has good effects*”

## 4. Discussion

As the results showed nurses applied various strategies to cope with job stress. Comparing the results of this study with other studies about coping of nurses, which were mostly quantitative and had used general inventories for assessment of coping methods, although shows the similarities, interesting finding of this study seems to have been less mentioned in other studies.

One of these cases was applying strategy named situational control of conditions by the researchers which include coping methods based on the working field of clinical nurses in which the nurses made attempts to control the situation through immediate curative and care measures or using procedures governing the organization as well as personal skills. Although, based on participants’ experiences, some of the coping methods such as leaving work with delay to ensure and control the working conditions come along with negative consequences such as physical and mental fatigue that the nurses expressed them while talking about their experiences. However, the results of the study conducted by Loo and Beh (2011) which had a quantitative-qualitative approach also showed (63%) of participants preferred to use the control mechanism in in job stressful situations. And this mechanism has allocated the highest frequency among other proposed mechanisms. Chang and et al. (2007) concluded that having control over the job plays the role of predictor of health in nurses, but Karasek (1990) had discovered it earlier that employees who have control over their tasks have better health status than those who have less control over their duties.

In fact, presumably, the people who have the ability to control the working conditions can easily cope with occupational stress and thus, experience less job stress and can demonstrate high levels of job performance. In the same way, the control-demand model, which was introduced by Karasek, is an interactional model which is between psychological demands and the threshold of decision-making (control). This model has two dimensions: the first dimension consists of job applications which are defined in terms of pressure, workloads, conflicts and confusions in responsibilities and required skills in a work environment. The second dimension of this model is the control which consists of two elements of decision-making power and direct skill (a range of skills). The highest pressure on this model is when the staff experience a lot of stress and demand while a small amount of control is available to cope with stressful situations (Karimi & Alipour, 2011).

But as was seen, the results showed that the participating nurses expressed the ways named preventive monitoring of situation to cope with job stress. Folkman and Moskowitz (2004) suggest that although the concept of risk-hurt or expected loss is the main cognitive theories of stress, many studies have focused on how to cope with the individuals’ present or past events. In fact, one of the new developments that have been achieved includes the ways in which people try to prevent the effects of events that are known as potential stressful factors and the responses have been introduced as the proactive coping.

Greenglass (2002) also believes that traditional forms of coping tend to have reactive coping in which the people behave according to the stressful events that have already occurred to compensate the loss or damage that has already occurred. But preventive coping is more prospective and included efforts to provide the public resources that improve the achievement of challenging objectives. The second difference between the reactive and preventive coping is that reactive coping is considered as the management of risk and preventive coping is objective management. In preventive coping, the people have the same viewpoints. They see risks, demands and opportunities in the future. They do not assess these issues as a threat, damage, or loss, but consider them as hard and challenging situations. Thus, preventive coping substitutes objective management instead of risk management.

However, a review of studies on how nurses cope with occupational stress which have been conducted primarily with quantitative and public tools of coping confirms that the proactive coping has not been reported as a coping strategy (Ahangarzadeh Rezaei, Shams, & SaghiZadeh, 2008; Akbarbeglu & ValiZadeh, 2011; Laal & Aliramaie, 2010; Lambert et al., 2004; Chang et al., 2007; Shiray, 2009; Mark & Smith, 2012; Beh & Loo, 2012). But this important issue has been proposed more as a coping strategy concerning the nurses’ occupational burnout and its positive achievements have been reported (Garcia, 2010; Gillespie & Gates, 2013; Chang & Chan, 2015). Thus preventive monitoring strategy is an interesting finding in this study, which emphasizes the role of context and using specific tools in assessment of strategies of coping with stress, the issue which has less been considered by the most researchers in studies of coping strategies of nurses.

The results of the study showed that the nurses, in some cases, benefit from some of the methods of seeking help strategy in encountering professional stressful situations and feeling stress arising from it. Comparing the results of this study with other studies on the subject of nurses coping with job stress indicates that, in other studies, like the present study, seeking help from others, or in other words, absorbing social supports has always been a strategy used by nurses to cope with the job stress. (Jahanshahi et al., 2014; Callaghan, Tak-Ying, & Wyatt, 2000; Makie, 2003; Lambert et al., 2004; Chang et al., 2006; Beh & Loo, 2012)

In some stressful situations, nurses used strategy named self-control and applying this strategy they tried to increase psychological and physical adaptation in emotional, physical, and cognitive aspects which situation cause the imbalance in them. This function, which proposes a very important aspect of self-control namely, self-regulation, overlaps the concept of coping based on the views of scholars such as (Compas, O’Connor-Smith, Saltzman, Thomsen, & Wadsworth, 2001; Eisenberg, Fabes, & Gutherie, 1997; Skinner, 1999; Skinner & Zimmer-Gembeck, 2007, 2009) as “regulation at stress”, although, besides self-controlling, coping tries to influence the stressful external environments and situations (Aldwin, Skinner, Zimmer-Gembeck, & Taylor, 2010) which was obvious in this study in the situational controlling of condition of nurses. Other results of studies dealing with the issue of nurses’ job stress also showed that self-control can be considered as one of the strategies of nurses to cope with job stress, which can point out to the studies conducted in this area (Jahanshahi et al., 2014; Lambert et al., 2004; Chang et al., 2007; Gholamzadeh, Sharif, & Dehghan Rad, 2011).

Although the results of this study suggest that some self-control methods, such as tolerance and forced acceptance by the nurses increase the stress in nurses, it would make unpleasant physical and psychological consequences for them and in some stressful situations the nurses preferred to avoid some stressful situations and totally reduce stress and achieve peace with taking distance from it in any way possible. This result also is similar to the results of some conducted studies, although the results of other studies show that nurses use this strategy not so much (Martins, Chaves, & Campos, 2014; Rodriguesi & Chaves, 2008).

The results of this study indicated that nurses use spiritual practices such as prayer to deal with some job stressful situations and using this strategy reduces tensions among nurses. Gall and et al. (2005) believed that similar to the concept of personality, spirituality has a complex and multidimensional structure, which is appeared in the process of behavior, beliefs and experiences of the individual. The multidimensionality of the structure causes that spirituality acts in several levels at a time. Spirituality can act at the level of individual factors (for example, individual opinions), primary and secondary assessments (accepting the power and authority of God), coping behaviors (for example, saying prayers and read the holy book), coping resources (for example, the relationship with nature), and meaning making (for example spiritual re-evaluation). The study of Jannati and et al. (2011), which was a qualitative study, showed that spiritual coping is a strategy used by clinical nurses to cope with job stress. In the study of Harris (2013), which was conducted on nurses working in hospices, use of prayer or meditation, along with social supports and sense of humor was one of three strategies for coping with job stress. Also, in the study of Bakibinga and et al. (2014), all nurses participating in the study stated that their religious values had positive effects on their performances and in challenging situations, their faith in God has helped them to cope with the situation better and even make them keep their jobs in hard situations.

## 5. Conclusion

Qualitative Investigation of nurses’ coping strategies with the stress caused by job stressors and contextual conditions governing the clinical field manifested some nurses’ coping strategies. It seems that the results of recent studies in which they used common tools of coping methods has less been considered as the result and this issue can bring more attention to the role of context, especially in studies related to nurses’ coping and conducting more qualitative research which require the ability to explore experiences of nurses and making clear their original strategies in encountering job stress. On the other hand, and according to the results, the planning and the adoption of necessary measures by the authorities to improve control and dominance of nurses on job stressful situations, boosting positive self-controlling methods, creating an atmosphere of cooperation and support and paying attention to spiritual growth among nurses to cope better with the professional stress is necessary and is probably effective in nurses’ adaptation with their jobs and promoting their health which are essential elements for achieving quality nursing care.
